# The clinical features of chronic intestinal schistosomiasis-related intestinal lesions

**DOI:** 10.1186/s12876-020-01591-7

**Published:** 2021-01-06

**Authors:** Xian Qin, Cai-Yuan Liu, Yi-Lin Xiong, Tao Bai, Lei Zhang, Xiao-Hua Hou, Jun Song

**Affiliations:** Division of Gastroenterology, Union Hospital of Tongji Medical College, Huazhong University of Science and Technology, 1277 Jiefang Road, Wuhan, 430022 China

**Keywords:** *Schistosoma japonicum*, Intestinal lesions, Case–control, Retrospective analysis, Colonoscopy

## Abstract

**Background:**

Chronic intestinal schistosomiasis has been reported to be associated with colonic polyps, colorectal cancer and ulcerative colitis. We aim to investigate the clinical characteristics of intestinal-related lesions caused by chronic intestinal schistosomiasis japonicum.

**Methods:**

Patients with and without chronic intestinal schistosomiasis were retrospectively enrolled from the endoscopy center of Wuhan Union Hospital from September 1, 2014, to June 30, 2019 with a ratio of 4:1. The characteristics of infected intestinal segments were analyzed in patients with chronic intestinal schistosomiasis. We also compared the characteristics of intestinal-related lesions, including colorectal polyps, colorectal cancer (CRC), ulceration or erosion of the intestinal mucosa and hemorrhoids, between the two groups.

**Results:**

A total of 248 patients with chronic intestinal schistosomiasis and 992 patients without chronic intestinal schistosomiasis were analyzed. The most common sites of chronic intestinal schistosomiasis were the sigmoid colon (79.0%) and rectum (84.7%). The frequency of intestinal polyps (64.5% vs. 42.8%, *p* < 0.001), especially rectal polyps (62.5% vs. 45.0%, *p* = 0.002), in the intestinal schistosomiasis group was significantly higher than that in the control group. Morphologically, type IIa polyps were more common in the schistosomiasis enteropathy group (68.5% vs. 60.7%, *p* = 0.001). Female patients with intestinal schistosomiasis had a higher detection rate of CRC than women in the control group (13.8% vs. 5.4%, *p* = 0.017). There was no significant difference in the incidence of ulcerative colitis between the two groups (0.8% vs. 0.6%, *p* = 0.664). In addition, the schistosomiasis enteropathy patients had a higher detection rate of internal hemorrhoids (58.9% vs. 51.0%, *p* = 0.027).

**Conclusions:**

Chronic intestinal schistosomiasis mainly involved the rectum and sigmoid colon and was more likely to induce intestinal polyps, especially rectal polyps and internal hemorrhoids. Women with chronic schistosomiasis have a higher risk of colorectal cancer.

## Background

Schistosomiasis is a serious zoonotic epidemic and infectious parasitic disease that can cause liver, spleen, gastrointestinal and many other organ lesions. As the place where all types of schistosoma grow and mature, liver fibrosis and atrophy gradually after long-time infection, forming a typical ‘‘pipestem fibrosis’’ change on ultrasound or pathological examination. Portal hypertension after liver atrophy leads to splenomegaly and hyperfunction [1]. Obstructive pulmonary hypertension is the main pathological feature of pulmonary schistosomiasis, further leading to pulmonary dilatation and right heart failure. Schistosome skin infections are uncommon and the mostly affected site is genital skin. Ocular lesions usually present as palpebral conjunctival nodules. While urinary schistosomiasis can lead to ureteral obstruction and chronic nephritis. Schistosomiasis of the central nervous system is mostly serious, and can form cerebral hernia, epilepsy, spinal cord and nerve root lesions, etc. [1, 2]. And the intestinal tract is a common infectious site of schistosoma.

The acute and chronic intestinal inflammation caused by *Schistosoma* is called intestinal schistosomiasis. The manifestations of acute schistosomiasis intestinal infection are intestinal mucosal hyperemia, edema and punctured hemorrhage. Microscopic examination reveals numerous neutrophils and eosinophil deposits. Patients present with fever, abdominal pain, diarrhea, stool with blood, etc. A few patients may present with symptoms of acute appendicitis due to noncalcified eggs deposited in the appendix [3, 4]. Chronic infection manifests as intestinal wall vascular network disorder, smooth or prominent mucous membrane yellow-white or grayish-yellow nodules, colonic polyps, and intestinal lumen stenosis. The yellow nodules are mostly the presentations of calcified worm egg deposition, fibrous tissue thickening and atrophy of the overlying mucosa. Lymphocytes and plasma cells can be seen under a microscope [3, 5, 6]. Patients may have intestinal obstruction, intussusception, abdominal mass and other symptoms [4]. Acute and chronic inflammatory changes coexist in the intestinal mucosal tissues of people in some epidemic areas due to repeated infection with *Schistosoma*, and their clinical symptoms are varied [3, 6, 7].

Many studies have suggested that chronic intestinal infection by *Schistosoma* is associated with colonic polyps, colorectal cancer and ulcerative colitis. There were very few comparative studies of large sample systems in the same period of intestinal lesions related to chronic schistosomiasis, and they were mainly concentrated on colonic polyps and colorectal cancer, while other intestinal lesions, such as mucosal ulcers, erosion and hemorrhoids, have not yet been studied. Therefore, we performed a systematic comparative analysis on the clinical characteristics of intestinal lesions associated with chronic schistosomiasis japonicum, including colorectal polyps, colorectal cancer, intestinal mucosal ulcer or erosion and hemorrhoids, through a large sample retrospective control study to provide a basis for the prevention and treatment of chronic intestinal schistosomiasis. We can clearly identify the subsequent diagnosis and treatment, follow-up observation of schistosomiasis patients who have been cured by observing and summarizing the overall involvement of schistosoma japonicum in colorectal tract, so as to detect intestinal benign and malignant diseases at an early stage. Early intervention of colorectal diseases caused by schistosoma can reduce the overall incidence and mortality of colon cancer, relieve the economic burden of patients and reduce pain to a certain extent.

## Methods

### Study design

This was a single retrospective case–control study. Patients with chronic schistosomiasis japonica were enrolled. Matched with the age and gender of the schistosomiasis group, the control group consisted of patients without schistosomiasis examined by our institution during the same time period, and the ratio of the control group to the schistosomiasis group was 4:1.

### Patients

Cases of chronic schistosomiasis japonica diagnosed in the endoscopy center of Union Hospital affiliated with Tongji Medical College of Huazhong University of Science and Technology were collected from September 1, 2014 to June 30, 2019. Inclusion criteria were in accordance with the diagnostic criteria of schistosomiasis (WS261-2006) issued by the Ministry of Health, PRC: (1) patient was from schistosomiasis endemic areas; (2) patient with or without diarrhea, abdominal pain and other symptoms, 3) calcified *Schistosoma* eggs identified by colorectal mucosal biopsy or HE staining. Exclusion criteria included the following: (1) prior history of intestinal surgery and/or endoscopic polypectomy; (2) previous diagnosis of familial adenomatous polyposis, hereditary nonpolyposis colorectal cancer, PJ syndrome, proliferative polyp syndrome or other hereditary colorectal cancer syndromes; (3) previous diagnosis of inflammatory bowel disease. Inclusion criteria for the control group were as follows: patients between 20 and 89 years old were collected according to the age distribution of the chronic schistosomiasis group. The exclusion criteria were as follows: confirmed or highly suspected schistosomiasis, (2) previous diagnoses of colorectal cancer and/or inflammatory bowel disease, (3) prior history of intestinal surgery and/or endoscopic polypectomy, and 4) previous diagnosis of the aforementioned hereditary colorectal cancer syndrome.

### Colonoscopy and sampling

Olympus colonoscopy was used during the examination (type: CF-H260AI, CF-H260AZI, CF-H290L/I, CF-HQ290L/I). The patient began a low-residue fluid diet 1 day before examination, and 4 bags of polyethylene glycol electrolyte powder were taken orally with 1 L of drinking water, 4 L in total. One bag each was taken orally at 8 and 9 p.m. the day before the inspection, one bag at 6 a.m. the day of inspection, and the last bag was taken orally 3 h before the colonoscopy. Simethicone oil (30 mL) was taken orally 1 h before examination for intestinal preparation. Intestinal cleansing prior to inspection was affirmed. Fecal sample without fecal matter were confirmed. Another bag of polyethylene glycol electrolyte powder should be added if the excretion is cloudy or contains stool. For the yellow nodules of the intestinal mucosa and other intestinal lesions suspected of schistosomiasis, biopsy forceps were used to take the tissues for microscopy. Biopsy was performed for intestinal polyps and bulges. Alternatively, argon plasma coagulation (APC), cold biopsy forceps, endoscopic mucosal resection (EMR), endoscopic submucosal dissection (ESD) and other endoscopic treatments were used to remove the lesions and sent for pathological examination. Multisite biopsy was performed for intestinal neoplasms. Biopsy of local mucosa was performed according to the degree of intestinal mucosa ulcer or erosion.

### Observation targets

The sex and age of the patients were recorded. The position, size, number, morphology and pathological type of colorectal polyps were observed. We also recorded the occurrence site of colorectal cancer, erosion or ulceration of the colon mucosa and hemorrhoids.

### Statistical analysis

SPSS 23.0 software was used for statistical analysis. Two or more constituent ratios are compared using the chi-square test or Fisher's exact test. Student's t test was used to compare the mean of two independent samples of normal distribution, and the data were expressed as “*M* ± *SD*”. The two independent samples of nonnormal distribution were analyzed using the Mann–Whitney U test, and the data were expressed as "median (lower quartile–upper quartile)". The correlation analysis of ranked data was conducted by Spearman's rank correlation test. *p* < 0.05 indicates statistical significance.

## Results

### General information

In total, 248 chronic intestinal schistosomiasis patients were enrolled, including 183 males and 65 females, with an average age of 60.5 ± 12.2 years. A total of 992 patients, including 732 males and 260 females, were included in the control group, and the average age was 57.8 ± 11.6 years old. The ratio of males to females in both groups was 2.82:1, with no significant difference in mean age (*p* = 0.164).

### Intestinal infection site of Schistosoma

**Fig. 1:**
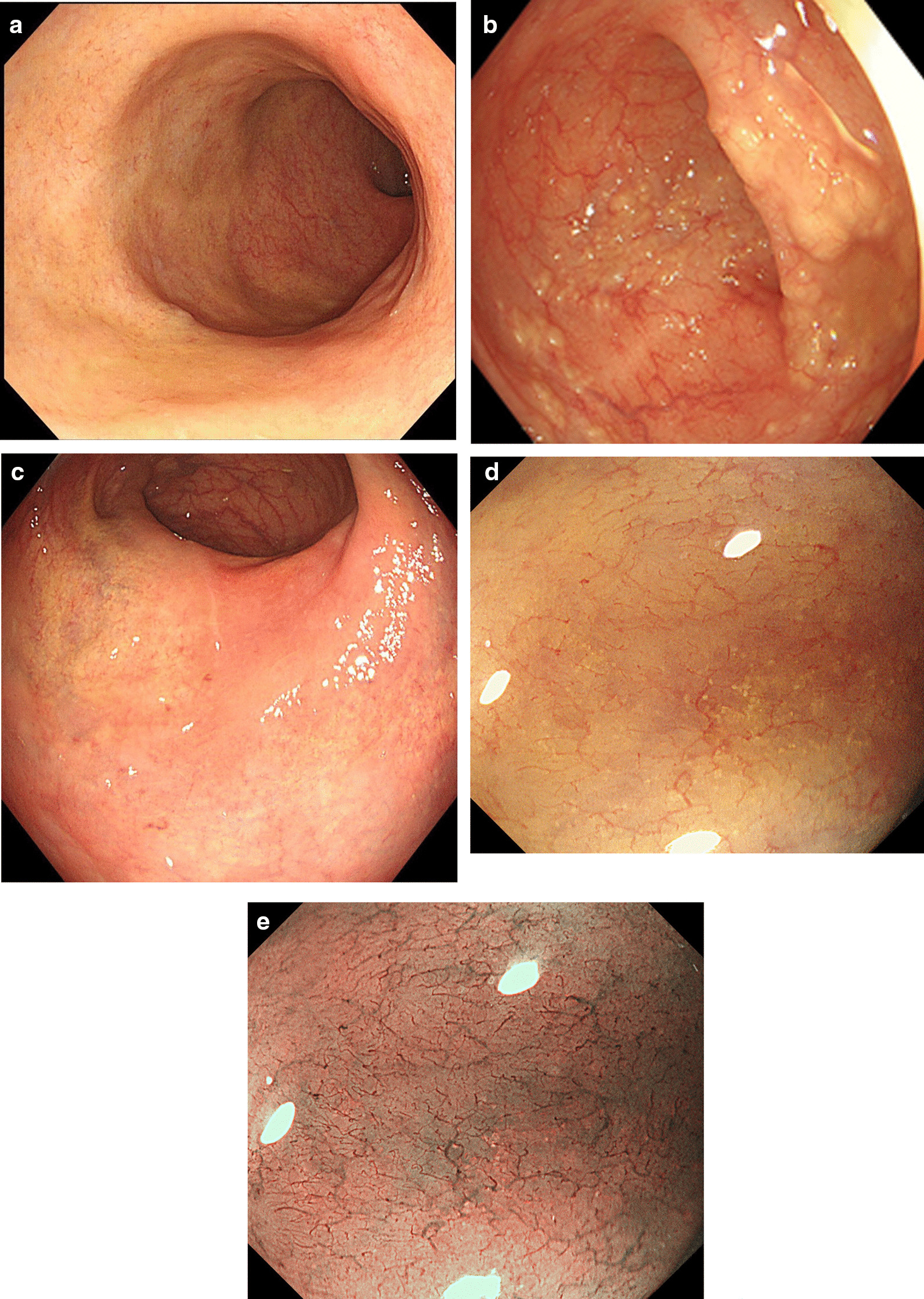
Colonoscopy: **a** Yellowish rectal mucosa. **b** Yellow miliary *Schistosoma* egg deposits in the sigmoid colon. **c** Yellow-white mucosal patch of the rectal mucosa. **d** Magnifying endoscopy shows yellow granular material with thickened, disordered, borderless submucosal vessels. **e** M-NBI: Unclear pit pattern with irregular submucosal vessels of varying thickness

**Fig. 2:**
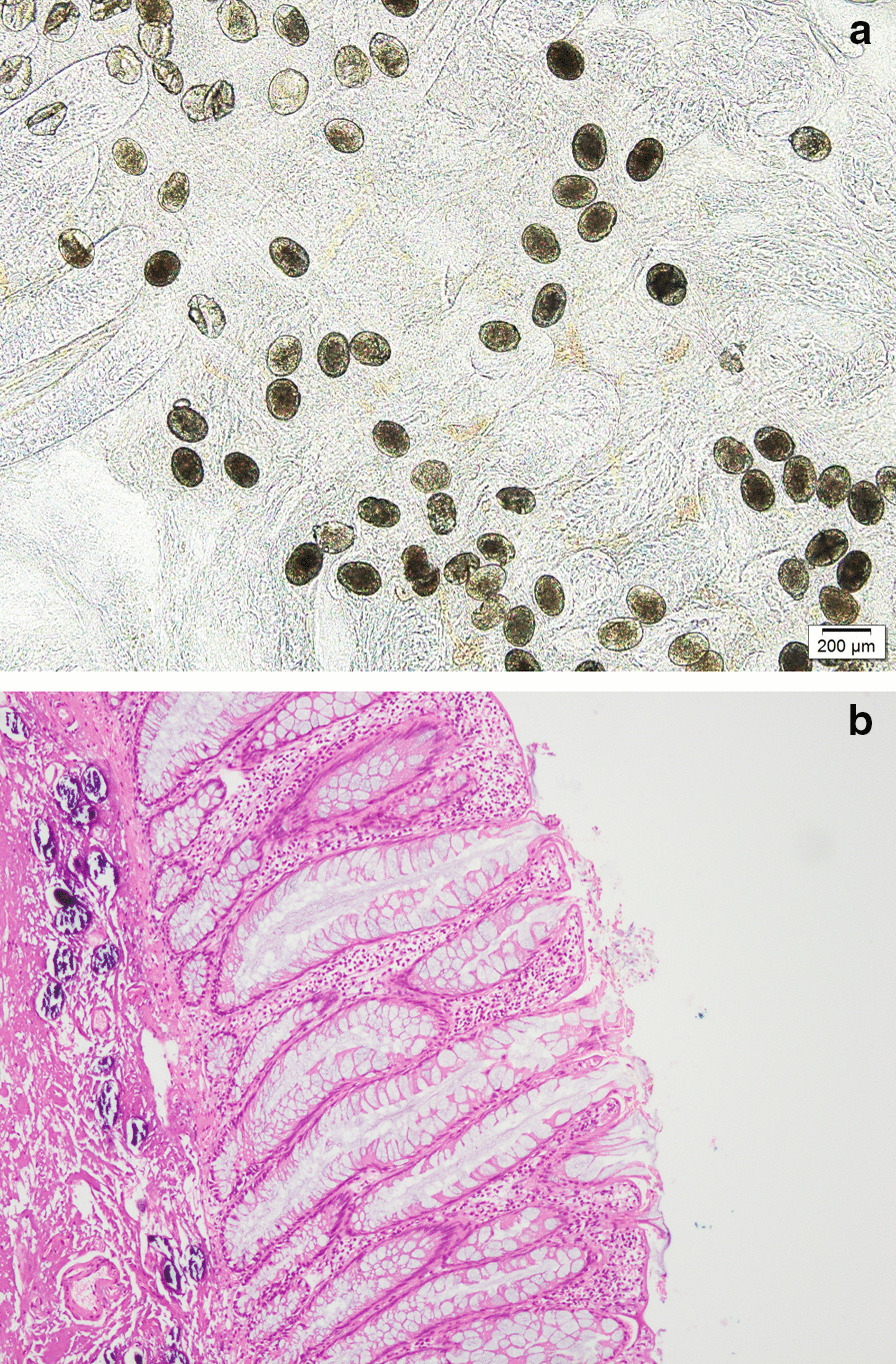
**a** Many *Schistosoma japonicum* eggs were deposited in the diseased intestinal mucosa. **b** HE staining showed many schistosomal egg deposits in the submucosa of the diseased tissue

The typical yellow nodules, miliary changes and microscopic appearance of the mucosa caused by schistosomiasis are shown in Figs. 1 and 2. The infectious intestinal segments of *Schistosoma* were 13.3% in the ileocecal region, 13.3% in the ascending colon, 12.5% in the transverse colon, 25.8% in the descending colon, 79.0% in the sigmoid colon, and 84.7% in the rectum. The proportion of the descending colon, sigmoid or rectum affected was 96.4%. Female patients were older than male patients (63.8 ± 9.9 y vs. 59.3 ± 12.7 y, *p* = 0.008) and had a higher rate of ileocecal involvement (10.4% vs. 21.5%, *p* = 0.023).

### The clinical features of intestinal polyps in the two groups

The details of the clinical characteristics of colorectal polyps between the two groups are shown in Table 1.

**Table 1:** Polyps in the two groups

	Patients with schistosomiasis	Patients without	*P* value^a^
	schistosomiasis (N = 248)	schistosomiasis (N = 992)	
Gender male/female	183/65	732/260	
Age	60.5 ± 12.2	57.8 ± 11.6	0.164
Polyps	160 (64.5%)	425 (42.8%)	< 0.001
Male/female	123 (67.2%)/37 (56.9%)	333 (45.5%)/92 (35.4%)	< 0.001/0.002
20–53 y	41 (55.4%)	113 (33.6%)	< 0.001
54–62 y	38 (66.7%)	122 (48.0%)	0.011
63–70 y	40 (65.6%)	134 (46.7%)	0.007
71–89 y	41 (73.2%)	56 (48.7%)	0.002
Site of polyps			
Ileocecal junction	13 (8.1%)	28 (6.6%)	0.516
Ascending colon	23 (14.4%)	70 (16.5%)	0.537
Transverse colon	43 (26.9%)	122 (28.7%)	0.661
Descending colon	31 (19.4%)	113 (26.6%)	0.071
Sigmoid colon	78 (48.8%)	224 (52.7%)	0.393
Rectum	100 (62.5%)	206 (48.5%)	0.002
Single/multiple segments	75 (46.9%)/85 (53.1%)	227 (53.4%)/198 (46.6%)	0.158
Number of polyps	2 (1–5)	2 (1–5)	0.153
Male/female	3 (1–7)/2 (1–3.25)	2 (1–6)/1 (1–3)	0.287/0.173
20–53 y	2 (1–4)	2 (1–3.5))	0.482
54–62 y	3 (2–6)	2 (1–5)	0.050
63–70 y	2 (1–8)	2 (1–6)	0.621
71–89 y	3 (1–4.5)	3 (1–6)	0.686
Multiple polyps	115 (71.9%)	272 (64.0%)	0.073
20–53 y	24 (58.5%)	63 (55.8%)	0.758
54–62 y	30 (78.9%)	77 (63.1%)	0.070
63–70 y	31 (77.5%)	93 (69.4%)	0.321
71–89 y	30 (73.2%)	39 (69.6%)	0.705
Morphology (n1 = 537, n2 = 1421)			
Is	141 (26.3%)	456 (32.1%)	0.012
Ip	7 (1.3%)	50 (3.5%)	0.009
Isp	14 (2.6%)	42 (3.0%)	0.680
IIa	368 (68.5%)	862 (60.7%)	0.001
IIb	4 (0.7%)	8 (0.6%)	0.746
IIc	0 (0.0%)	0 (0.0%)	/
LST	3 (0.6%)	3 (0.4%)	0.401
Size (n1 = 662, n2 = 1639)			
1–5 mm	597 (90.2%)	1451 (88.5%)	0.252
6–9 mm	48 (7.3%)	122 (9.4%)	0.873
10–19 mm	13 (2.0%)	53 (3.2%)	0.099
≥ 20 mm	4 (0.6%)	13 (0.8%)	0.632
Pathology (n1 = 249, n2 = 487)			
Serrated lesions and polyps	112 (45.0%)	244 (50.1%)	0.188
Hyperplastic polyp	111 (99.1%)	240 (98.4%)	1.000
Traditional serrated adenoma	1 (0.9%)	2 (0.8%)	1.000
Serrated lesions with dysplasia	0 (0.0%)	2 (0.8%)	0.552
Traditional adenoma	137 (55.0%)	243 (49.9%)	0.188
Tubular adenoma	125 (91.2%)	223 (91.8%)	0.859
villous adenoma	1 (0.7%)	2 (0.8%)	1.000
Tubulovillous adenoma	11 (8.0%)	18 (7.4%)	0.826
Number of adenomas	2 (1–4)	2 (1–3)	0.225
Male/female	3 (1–4)/1 (1–2)	2 (1–3)/1 (1–1)	0.210/0.496

^a^Use student's *t* test to compare the age of the two groups. Median and 25th–75th percentile describe the number of polyps or adenomas in different age groups and gender. Categorical values compared with chi-square test (polyp detection, site of polyps, multiple polyps, morphology, pathology), and Fisher's exact test was used when expected count of one set of samples was less than 5(IIb, LST, ≥ 20 mm, traditional serrated adenoma, serrated lesions with dysplasia, villous adenoma)

#### Polyp detection rate

The overall polyp detection rate (PDR) of patients with schistosomiasis was 64.5%. According to the number of patients, the schistosomiasis group was divided into five age groups: 20–48 years old, 49–57 years old, 58–64 years old, 65–71 years old, and 72–89 years old. Each group had a roughly equal number of patients. The PDR of the above age group was 55.1%, 58.3%, 64.6%, 69.8% and 74.0%, respectively, showing a gradually increasing trend (*p* = 0.022). The number of polyps was positively correlated with age (*p* = 0.002).

The PDR of intestinal schistosomiasis patients was significantly higher than that of the control group (64.5% vs. 42.8%, *p* < 0.001). The frequency of polyps in both male and female schistosomiasis intestinal patients was higher than that in the same-sex people in the control group (male: 67.2% vs. 45.5%, *p* < 0.001; female: 56.9% vs. 35.4%, *p* = 0.002). People were divided into four age groups, including 20–53 years old, 54–62 years old, 63–70 years old, and 71–89 years old, according to the status of polyps detected in the schistosomiasis group. The polyp case number in every age range of the schistosomiasis group was similar. The results showed that the PDR of the schistosomiasis group was higher than that of the control group (20–53 y: 55.4% vs. 33.6%, *p* < 0.001; 54–62 y: 66.7% vs. 48.0%, *p* = 0.011; 63–70 y: 65.6% vs. 46.7%, *p* = 0.007; 71–89 y: 73.2% vs. 48.7%, *p* = 0.002).

#### Location of polyps

The frequency of rectal polyps was higher in the schistosomiasis group (62.5% vs. 48.5%, *p* = 0.002). No significant differences were found for the incidence of polyps in the ileocecal region, ascending colon, transverse colon, descending colon and sigmoid colon between the two groups (ileocecal region: 8.1% vs. 6.6%, *p* = 0.516; ascending colon: 14.4% vs. 16.5%, *p* = 0.537; transverse colon: 26.9% vs. 28.7%, *p* = 0.661; descending colon: 19.4% vs. 26.6%, *p* = 0.071; sigmoid colon: 48.8% vs. 52.7%, *p* = 0.393).

#### Number of polyps

The proportion of multiple polyps in the schistosomiasis group was 71.9%, which was higher than 64.0% in the control group, but the difference was not statistically significant (*p* = 0.073). No significant differences were found in the number of polyps between the two groups of men and women (3 (1–7) vs. 2 (1–6), *p*1 = 0.287; 2 (1–3.25) vs. 1 (1–3), *p*2 = 0.173). There was also no significant difference in the number of polyps in different age groups (*p*1 = 0.482, *p*2 = 0.050, *p*3 = 0.621, *p*4 = 0.686). However, the number of polyps in intestinal schistosomiasis patients aged 54 to 62 years was higher than that in the control group, with marginal statistical significance (3 (2–6) vs. 2 (1–5), *p* = 0.050) (Table 1).

#### Morphology of polyps

**Fig. 3:**
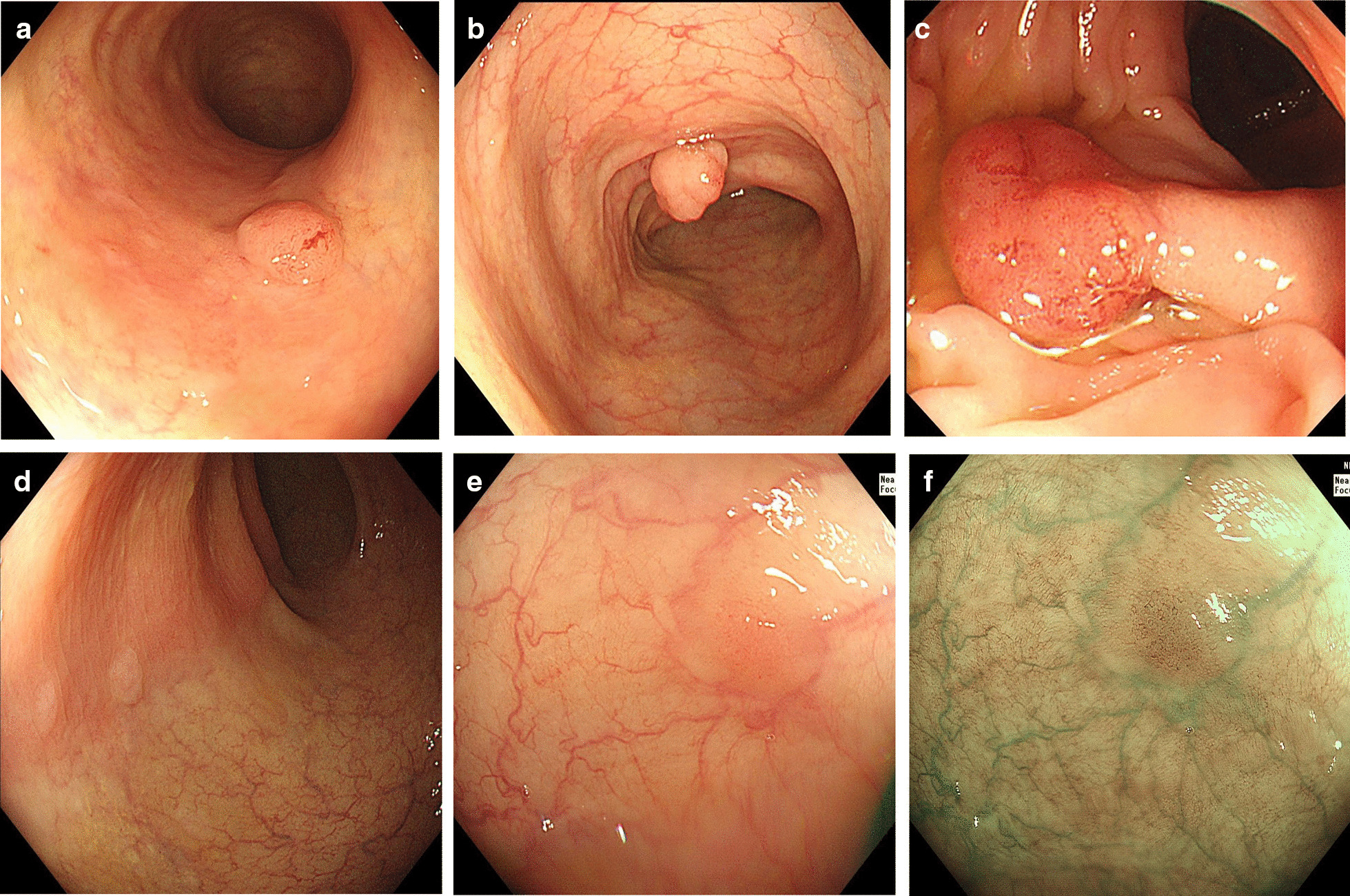
Polyps of different morphologies in patients with chronic schistosomiasis: type Is (**a**), Isp (**b**), Ip (**c**), IIa (**d**) and IIb (**e**, **f**) polyps

The morphology of the colorectal polyps was classified into three types according to the Paris classification [8]. Various types of colon polyps in the schistosomiasis group are shown in Fig. 3. There were 537 polyps in the schistosomiasis group and 1421 polyps in the control group. The proportion of type IIa polyps in the schistosomiasis group was higher than that in control group (68.5% vs. 60.7%, *p* = 0.001), but the number of type Is and Ip polyps was less than the control group (Is: 26.3% vs. 32.1%, *p* = 0.012. Ip: 1.3% vs. 3.5%, *p* = 0.009). The proportion of type Isp polyps in the two groups was 2.6% and 3.0%, without a significant difference (*p* = 0.680). The proportion of type IIb polyps in the two groups was 0.7% and 0.6%, respectively (*p* = 0.746). In addition, there were 3 laterally spread tumors in each of the two groups (*p* = 0.401).

#### Size of polyps

A total of 662 polyps were found in the schistosomiasis group, and 1639 colon polyps were counted in the control group in our study. There was no significant difference in the proportion of 1–5 mm polyps between the schistosomiasis group and the control group (90.2% vs. 88.5%, *p* = 0.235). The proportions of polyps of 6–9 mm, 10–19 mm and ≥ 20 mm in the two groups were roughly the same (6–9 mm: 7.3% vs. 9.4%, *p* = 0.873. 10–19 mm: 2.0% vs. 3.2%, *p* = 0.099. ≥ 20 mm: 0.6% vs. 0.8%, *p* = 0.632).

#### Pathology of polyps

The pathological type of 249 polyps in the schistosomiasis group and 487 polyps in the control group was confirmed. The proportion of patients with polyp pathological results in the two groups was 41.3% and 35.5%, with no significant difference (*p* = 0.202).

There were no significant differences in the overall pathological classification between the two groups (serrated lesions and polyps: 45.0% vs. 50.1%, adenomas: 55.0% vs. 49.9%, *p* = 0.188). Two traditional serrated adenomas (TSAs) and 2 serrated lesions with dysplasia were detected in patients without schistosomiasis. Only 1 TSA was detected in the schistosomiasis group, but no serrated lesions with dysplastic polyps were found. The proportion of tubular adenomas in the schistosomiasis group was approximately 91.2%, with no significant difference compared with the control group (91.8%, *p* = 0.859). There was no significant difference in the proportion of tubular villous adenomas (8.0% vs. 7.4%, *p* = 0.826). In addition, one villous adenoma was detected in the schistosomiasis group and two in the control group.

The proportion of traditional adenoma in intestinal schistosomiasis patients aged 71 to 89 years was higher than that in the control group (35.0% vs. 11.4%, *p* < 0.001), but no significant difference was found in the other age groups (20–53 y: 14.3% vs. 11.1%, *p* = 0.334; 54–62 y: 20.5% vs. 19.8%, *p* = 0.842; 63–70 y: 14.6% vs. 13.8%, *p* = 0.781).

### Clinical features of colorectal cancer

A total of 19 intestinal schistosomiasis patients were diagnosed with CRC by pathological biopsy with an average age of 69.2 ± 13.4 years, which was greater than that of the control group (*p* = 0.016). There was no significant difference in the detection rate of CRC between the two groups (7.7% vs. 5.7%, *p* = 0.261). However, the detection rate of CRC in women with schistosomiasis was higher than that of women in the control group (13.8% vs. 5.4%, *p* = 0.017). Male patients in both groups had similar detection rates for CRC (5.5% vs. 5.9%, *p* = 0.832) (Table 2).

**Table 2:** Colorectal cancer, hemorrhoids and intestinal mucosa ulcer or erosion in the two groups

	Patients with schistosomiasis (N = 248)	Patients without schistosomiasis (N = 992)	*p* Value^a^
Gender Male/Female	183/65/65	732/260	
Age	60.5 ± 12.2	57.8 ± 11.6	0.164
Colorectal cancer	19 (7.7%)	57 (5.7%)	0.261
Age	69.2 ± 13.4	67.5 ± 9.7	0.016
Male/female	10 (52.6%)/9 (47.4%)	43 (75.4%)/14 (24.6%)	0.832/0.017
Site			
Colon	12 (63.2%)	26 (45.6%)	0.185
Rectum	6 (31.6%)	26 (45.6%)	0.283
Colon and rectum	1 (5.3%)	5 (8.8%)	1.000
Left colon	12 (63.2%)	48 (84.2%)	1.000
Right colon	7 (36.8%)	8 (14.0%)	0.031
Entire colon	0 (0.0%)	1 (1.8%)	/
Scattered ulcer/erosion	22 (8.9%)	71 (7.2%)	0.359
Age	57.5 ± 13.0	57.5 ± 12.7	0.709
Male/female	11 (6.0%)/11 (16.9%)	44 (6.0%)/27 (10.4%)	1.000/0.142
Site			
Ileocecal junction	7 (32.4%)	20 (28.2%)	0.835
Ascending colon	2 (8.7%)	6 (8.5%)	1.000
Transverse colon	0 (0.0%)	7 (9.9%)	0.188
Descending colon	1 (4.3%)	17 (23.9%)	0.063
Sigmoid colon	6 (26.1%)	20 (28.2%)	0.846
Rectum	11 (47.8%)	45 (63.4%)	0.186
Ulcerative colitis	2 (0.8%)	6 (0.6%)	0.664
Hemorrhoids	149 (60.1%)	519 (52.3%)	0.028
Age	60.9 ± 11.5	57.7 ± 11.3	0.527
Male/female	112 (61.2%)/37 (56.9%)	369 (50.4%)/150 (57.7%)	0.009/0.911
Internal hemorrhoids	146 (58.9%)	506 (51.0%)	0.027
External hemorrhoids	16 (6.5%)	82 (8.3%)	0.343
Mixed hemorrhoids	13 (5.2%)	69 (7.0%)	0.331

^a^Used student's *t* test to compare the age of the two groups. Categorical values compared with chi-square test (colorectal cancer, scattered ulcer/erosion, hemorrhoids), and Fisher's exact test was used when expected count of one set of samples was less than 5(ulcerative colitis, colon and rectum cancer, scattered ulcer/erosion of ascending colon, transverse colon and descending colon)

### Clinical features of erosion or ulceration

**Fig. 4:**
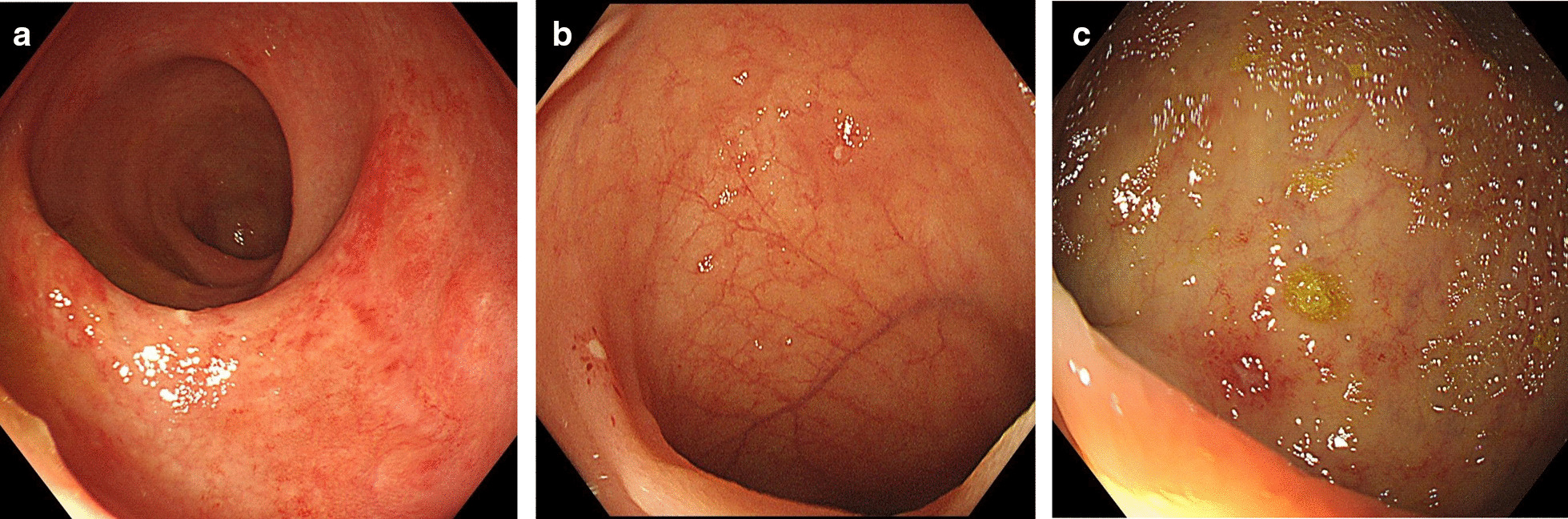
**a** Flaky erosion in the rectum of intestinal schistosomiasis patients. **b** Multiple ulcerations were observed in the terminal ileum after *Schistosoma* infection. **c** The rectum of chronic schistosomiasis patients had scattered shallow ulcers and erosion

In this study, a total of 22 intestinal schistosomiasis patients and 71 patients in the control group were found to have scattered ulceration or erosion during colonoscopy (Fig. 4). There was no significant difference in the detection rate of scattered ulceration or erosion of the intestinal mucosa between the two groups (8.9% vs. 7.2%, *p* = 0.359). The proportion of ulceration or erosion in the transverse colon, descending colon and rectum in intestinal schistosomiasis patients was lower than that in the control group, but without statistical significance (0.0% vs. 9.9%, *p*1 = 0.188. 4.3% vs. 23.9%, *p*2 = 0.063. 47.8% vs. 63.4%, *p*3 = 0.186). There was no significant difference in the proportion of ileocecal, ascending colon and sigmoid colon between the two groups (ileocecal colon: 32.4% vs. 28.2%, *p* = 0.835; ascending colon: 8.7% vs. 8.5%, *p* = 1.000; sigmoid colon: 26.1% vs. 28.2%, *p* = 0.846).

In addition, the endoscopic performance of 2 patients in the schistosomiasis group was highly consistent with ulcerative colitis, while 6 patients in the control group showed no significant difference (0.8% vs. 0.6%, *p* = 0.664) (Table 2).

### Clinical features of hemorrhoids

The detection rate of hemorrhoids in schistosomiasis patients was 60.1%, higher than 52.3% in the control group (*p* = 0.028). The detection rates of internal hemorrhoids in the two groups were 58.9% and 51.0%, respectively (*p* = 0.027). There was no significant difference in the detection rate of external hemorrhoids and mixed hemorrhoids (external hemorrhoids: 6.5% vs. 8.3%, *p* = 0.343; mixed hemorrhoids: 5.2% vs. 7.0%, *p* = 0.331). The detection rate of hemorrhoids in male schistosomiasis patients was higher than that in the control group (61.2% vs. 50.4%, *p* = 0.009). Women in both groups had roughly equal rates of hemorrhoids (56.9% vs. 57.7%, *p* = 0.911) (Table. 2).

## Discussion

### The detection method of intestinal schistosomiasis

Intestinal schistosomiasis is usually diagnosed by colonoscopy. Usually, intestinal mucosal characteristics under colonoscopy, pathological results of mucosal biopsy or microscopic examination are the main methods to diagnose it. Conventional abdominal CT has limited diagnostic efficacy in schistosomiasis. While virtual CT colonoscopy is a non-invasive test with an 86.7% sensitivity to polyps, CRC, inflammatory bowel disease or other colorectal diseases, and may play a certain role in intestinal diseases associated with chronic schistosomiasis [9]. In addition, diffusion-weighted imaging also has positive effects on determination of activity in Crohn's disease [10].

### Schistosoma infection and colorectal polyps

The distribution of schistosomiasis has obvious regional and sex characteristics. The ratio of intestinal schistosomiasis among men and women was 2.82:1. The number of male infections was significantly higher than that of females, which may be related to the higher number of opportunities for male exposure to contaminated water.

Many eggs were deposited in loose connective tissue in the submucosa of the intestinal wall after *Schistosoma* infection. Due to inflammation and foreign body reaction, tissue necrosis and granulomas formed in the deposition site of the eggs, which then stimulated the proliferation of fibrous tissue and the thickening of the mucosal muscle layer, resulting in yellow nodular changes and vascular network disorder visible to the naked eye under colonoscopy [5]. In our study, all schistosomiasis patients had different degrees of yellow intestinal mucosa, yellow flat or protruding nodules or vascular network disorder, and schistosomal eggs were observed under microscopy. Previous studies have shown that every segment of the intestinal tract of schistosomiasis patients can be involved, but the rectum, sigmoid colon and descending colon supplied by the inferior mesenteric vein were more common, accounting for 90% [1]. Our analysis showed that the rectum and sigmoid colon were the most common intestinal involvement sites of schistosomiasis, and 96.4% of the patients were infected in the descending colon, sigmoid colon or rectum, which was consistent with the above conclusions.

There were 160 cases of intestinal schistosomiasis with intestinal polyps. There was no significant difference in the detection rate of polyps between men and women. However, the incidence of polyps increased with age (*p* < 0.05). Previous studies have shown that the incidence of polyps in intestinal schistosomiasis patients is in direct proportion to age, and patients over 60 years old with colorectal polyps account for 91.25% of the total samples [11]. Our study revealed that the proportion of polyp patients over 60 years old was 59.4%.

The detection rate of polyps in chronic intestinal schistosomiasis patients was higher than that in the control group (*p* < 0.05), and rectal polyps were dominant (*p* = 0.002), which was consistent with the main sites of schistosomiasis infection, indicating that patients with chronic schistosomiasis intestinal infection had a higher risk of colon polyps. Recently, some scholars compared the intestinal polyp characteristics of patients with intestinal schistosomiasis to nonschistosomiasis patients through a retrospective study. The results showed that polyps less than 10 mm and sessile polyps were common in both groups [11]. In our study, the size of polyps in the two groups was also less than 10 mm, accounting for more than 90%. The morphology of polyps was mainly superficial bulges (type IIa) in the two groups (*p* > 0.05).

Our study showed that the proportion of traditional adenoma in intestinal schistosomiasis patients aged 71–89 years was higher than that in patients without schistosomiasis (*p* < 0.001). Therefore, patients exposed to chronic intestinal schistosomiasis may have a higher risk of adenoma. Xiao et al. compared the oncogene expression of schistosomiasis adenomatous polyps, schistosomiasis nonadenomatous polyps and colorectal cancer and found that the expression rate of protooncogenes such as Bcl-2 and CK–20 was higher in adenomatous polyps than in nonadenomatous polyps, and the expression rate of the tumor suppressor gene p27 was significantly lower (*p* < 0.001). The expression rates of CK-20 and p27 in adenomatous polyps were not significantly different from those in colorectal cancer tissues. Therefore, they believe that adenomatous polyps with schistosomiasis infection are an important factor for canceration [12].

### Schistosoma infection and carcinoma

We found that the combination rate of CRC in women with schistosomiasis was significantly higher than that in the control group (*p* = 0.017), but there was no difference in the overall detection rate of CRC between the two groups, indicating that chronic schistosomiasis may increase the incidence of colorectal cancer in women. However, the sample size of this study is relatively small, and larger studies are needed to determine whether the conclusion is correct.

Many studies have shown that schistosomiasis is closely related to colorectal cancer. About 6.3% to 37.1% of patients with colonic schistosomiasis japonica eventually develop colorectal cancer [13]. *S. japonicum* infection is considered an important risk factor for CRC and can increase mortality in CRC patients [1, 14]. The canceration mechanism of schistosomiasis is not fully understood, and inflammation may be the key factor in cancer [13, 15].

Liu et al. retrospectively analyzed 179 cases of intestinal schistosomiasis japonicum disease, among which 32 cases were complicated with colorectal cancer, and concluded that intestinal schistosomiasis was related to the incidence of CRC [16]. Wang et al. compared 30 patients with schistosomiasis rectal cancer (SRC) who underwent laparoscopic total mesorectal resection to 60 CRC patients without schistosomiasis. Multivariate analysis showed that schistosomiasis affected the disease-free survival and overall survival of rectal cancer patients, and the prognosis of SRC was considered to be worse than that of nonschistosomiasis rectal cancer (NSRC) [17]. However, Weng et al. compared 1,800 hot spot mutations in 22 genes of 26 schistosomiasis patients with CRC and 42 patients with simple colorectal cancer by targeted second-generation sequencing technology and found no significant difference between the two groups. They believed that schistosomiasis infection was less correlated with colorectal cancer, but the sample size was small [18].

CRC with chronic schistosomiasis is mostly adenocarcinoma [13]. Canepa et al. reported the first case of rectal signet-ring cell carcinoma [19]. Studies have reported that the average age of CRC patients with schistosomiasis is 6 to 16 years younger than that of people without schistosomiasis [13, 20]. However, some studies have shown the opposite [21]. A study of 80 schistosomiasis CRC patients and 258 nonschistosomiasis CRC patients showed that CRC patients with schistosomiasis infection were older (62.2 ± 9.6 y vs. 57.2 ± 11.7 y, *p* = 0.000) [22]. In our study, the average age of CRC patients with schistosomiasis was 69.2 ± 13.4 years, which was higher than that of people without schistosomiasis (*p* = 0.016).

### Schistosoma infection and colitis

Ulcerative colitis usually presents as extensive and consecutive erosion and multiple shallow ulcerations of the colon, covered with secretions, accompanied by hyperemia, edema or bleeding of surrounding tissue under the endoscope. We compared the erosion and ulceration of the intestinal mucosa between schistosomiasis patients and nonschistosomiasis patients. The ratio of ulceration or erosion in the transverse colon, descending colon and rectum of intestinal schistosomiasis patients was lower than that in the control group, with a large difference, but without statistical significance (*p* > 0.05). There was no significant difference in the detection rate of ulcerative colitis between the two groups (*p* > 0.05). We indicated that chronic schistosomiasis infection of the intestine has little influence on the incidence of ulcerative colitis, but it may reduce the risk of scattered ulceration or erosion of the colonic mucosa. A larger sample size is needed for further study.

Previous studies have shown that the incidence of inflammatory bowel disease (IBD) in epidemic areas of parasitic diseases is lower than in other regions [23]. An animal study demonstrated that the deposition of *S. japonicum* eggs prevented 5% 2,4,6-trinitrobenzene sulfonic acid (TNBS)-induced colitis in mice, which may be related to Th1/2 balance and regulation of Toll-like receptor 4 [24]. Liu et al. found in a mouse model that *S. japonicum* infection could prevent dextran sulfate sodium-induced colitis. The reduction in the inflammatory response is related to the Th1/Th2/Th17 pathway, NF-κB pathway and endoplasmic reticulum stress [25]. Wu et al. found that the soluble antigen of adult *S. japonicum* and recombinant cysteine protease inhibitor protein can alleviate colitis by promoting the immune response mediated by Treg and Th2 and suppressing the Th1 response in mice induced by TNBS [26]. Many animal studies tend to believe that schistosomiasis plays a protective role in IBD. Our study compared general performance, such as ulcers and erosion of the intestinal mucosa, under colonoscopy but lacked research at the pathological and molecular levels.

### Schistosoma infection and hemorrhoids

Clinically, internal hemorrhoids usually appear as the mass of varicose veins above the anorectal odontoid line, where the superior rectal vein and the middle rectal vein respectively drain the blood to the portal vein and the systemic vein [27]. As we know above, schistosomiasis mainly lives in the portal vein and its branches, especially the submesenteric vein, and thus can further reach the superior rectal vein. Therefore, we speculated that the formation of internal hemorrhoids may be related to the chronic inflammation caused by the stimulation of eggs, which aggravates the local venous reflux and affects the structure of the anal cushion. In addition, intestinal symptoms such as diarrhea and bloody stool caused by schistosomiasis may also promote the development of internal hemorrhoids. More research is needed.

## Conclusion

Schistosomiasis is still a global public health problem that urgently needs to be solved. The related diseases of schistosomiasis deserve attention. Chronic schistosomiasis mainly affects the rectum and sigmoid colon. Compared with patients without schistosomiasis, chronic intestinal schistosomiasis patients have a higher incidence of colorectal polyps, mainly rectal polyps. The detection rate and number of polyps gradually increased with age. Previous schistosomiasis infections may increase the risk of colorectal cancer in women. Chronic intestinal schistosomiasis patients have a higher incidence of internal hemorrhoids, but more research is needed to determine whether the two are linked.

## Data Availability

The datasets used or analysed during the current study are available from the corresponding author on reasonable request.

## Abbreviations

PRC: People's Republic of China
M-NBI: Magnifying Endoscopy with Narrow-Band Imaging
CRC: Colorectal cancer
APC: Argon plasma coagulation
EMR: Endoscopic mucosal resection
ESD: Endoscopic submucosal dissection
PDR: Polyp detection rate
TSA: Conventional serrated adenoma
SRC: Schistosomiasis rectal cancer
NSRC: Nonschistosomiasis rectal cancer
IBD: Inflammatory bowel disease
TNBS: 5% 2,4,6-Trinitrobenzene sulfonic acid

## References

[1] Barsoum RS, Esmat G, El-Baz T (2013). Human schistosomiasis: clinical perspective: review. J Adv Res.

[2] Abdel Razek AAKWA, Castillo M. Parasitic diseases of the central nervous system. Neuroimag Clin N Am. 2011;21:815–41. 10.1016/j.nic.2011.07.005 22032501

[3] Ye C, Tan S, Jiang L, Li M, Sun P, Shen L, Luo H (2013). Endoscopic characteristics and causes of misdiagnosis of intestinal schistosomiasis. Mol Med Rep.

[4] Nancy F, Crum HMC, Michael AF, Braden RH. Gastrointestinal Schistosomiasis japonicum Infections in Immigrants from the Island of Leyte, Philippines. J Travel Med. 2003;10:131–2. 10.2310/7060.2003.31779 12650659

[5] Elbaz T, Esmat G (2013). Hepatic and intestinal schistosomiasis: review. J Adv Res.

[6] Cao J. Endoscopic findings and clinicopathologic characteristics of colonic schistosomiasis: a report of 46 cases. World J Gastroenterol. 2010;16:6. 10.3748/wjg.v16.i6.723 PMC2817060 20135720

[7] Guo J, Shen L, Shen ZX, Tan SY, Luo HS: Endoscopic and hispathological characteristics of the intestinal schistosomiasis. Chin J Dig Endoscopy. 2006.

[8] Rlsekmvjiag T. Pragmatic classification of superficial neoplastic colorectal lesions. Gastroint Endoscopy. 2009;70(6):1182–99. 10.1016/j.gie.2009.09.015 19879563

[9] Razek AAA, Zeid MMA, Bilal M, Wahab NMA (2005). Virtual CT colonoscopy versus conventional colonoscopy: a prospective study. Hepatogastroenterology.

[10] Ahmed AA, Fahmy DM. Diagnostic value of diffusion-weighted imaging and apparent diffusion coefficient in assessment of the activity of Crohn disease: 1.5 or 3 T. J Comput Assist d Tomogr. 2018;2018:42. 10.1097/RCT.0000000000000754 PMC6296832 29958199

[11] Yang XH, Tan PS, Liu XL, Zheng F, Zhang SY, Xu M (2018). Clinical characteristics, diagnosis and treatment of colonic polyps with and without schistosomiasis. Chin J Control End Dis.

[12] Xiao J, Deng CS, Yi FM, Zhou JY (2013). Risk assessment of intestinal schistomiasis polyp progress to clolorectal carcinoma. Med. J. Wuhan Univ..

[13] Hamid HKS (2019). *Schistosoma japonicum*—associated colorectal cancer: a review. Am J Trop Med Hyg.

[14] Feng H, Lu AG, Zhao XW, Han DP, Zhao JK, Shi L, Schiergens TS, Lee SM, Zhang WP, Thasler WE (2015). Comparison of non-schistosomal rectosigmoid cancer and schistosomal rectosigmoid cancer. World J Gastroenterol.

[15] OE HS, Hamid HK, Mekki SO, Suleiman SH, Ibrahim SZ. Colorectal carcinoma associated with schistosomiasis: a possible causal relationship. World J Surg Oncol. 2010;8:68. 10.1186/1477-7819-8-68 PMC2928231 20704754

[16] Liu W, Zeng HZ, Wang QM, Yi H, Tang CW (2013). Schistosomiasis combined with colorectal carcinoma diagnosed based on endoscopic findings and clinicopathological characteristics: a report on 32 cases. Asian Pac. J. Cancer Prevent. Apjcp.

[17] Wang M, Zhang YC, Yang XY, Wang ZQ (2014). Prognostic analysis of schistosomal rectal cancer. Asian Pac J Cancer Prev.

[18] Weng J, Sun Y, Wen F, Zhou Z, Yin KL, Shen ED, Liu CY, Xie WT (2018). Gene mutation of patients with colorectal cancer combind schistomiasis. Chin. J. Cancer Prevent. Treat..

[19] Canepa M, Fanta PT, Weidner N, Peterson MR (2012). Schistosomiasis and signet ring cell carcinoma of the rectum. Ann Diagn Pathol.

[20] Yosry A (2006). Schistosomiasis and Neoplasia. Contrib Microbiol.

[21] Madbouly KM, Senagore AJ, Mukerjee A, Hussien AM, Shehata MA, Navine P, Delaney CP, Fazio VW (2007). Colorectal cancer in a population with endemic Schistosoma mansoni: is this an at-risk population?. Int J Colorectal Dis.

[22] Chen YB, Liu Z, Qian J, Feng HY, Li DC, Fan YT (2016). Expression difference of DNA mismatch repair gene hMLH1 and hMSH2 between schistomiasis-associated colorectal cancer and sporadic colorectal cancer. Chin J Gastroint Surg.

[23] Varyani F, Fleming JO, Maizels RM (2017). Helminths in the gastrointestinal tract as modulators of immunity and pathology. Am J Physiol Gastrointest Liver Physiol.

[24] Imai J, Ichikawa H, Mizukami H, Suzuki T, Watanabe N, Mine T (2016). Colonic high-grade tubular adenomas associated with *Schistosoma japonicum*. Tokai J Exp Clin Med.

[25] Liu Y, Ye Q, Liu YL, Kang J, Chen Y, Dong WG (2017). *Schistosoma japonicum* attenuates dextran sodium sulfate-induced colitis in mice via reduction of endoplasmic reticulum stress. World J Gastroenterol.

[26] Wu Y, Li L, Xu YW, Xing RX, Hu J, Wang SS, Shen JL, Xu YH, Chen X (2019). *Schistosoma japonicum* soluble worm proteins and recombinant cystatin ameliorate experimental colitis in a murine model. Chin J Parasitol Parasit Dis.

[27] Margetis N (2019). Pathophysiology of internal hemorrhoids. Ann Gastroenterol.

